# A single-arm phase II trial of weekly nanoparticle albumin-bound paclitaxel (nab-paclitaxel) monotherapy after standard of chemotherapy for previously treated advanced non-small cell lung cancer

**DOI:** 10.1007/s00280-019-03843-0

**Published:** 2019-04-16

**Authors:** Yasuhiro Kato, Yusuke Okuma, Kageaki Watanabe, Makiko Yomota, Shoko Kawai, Yukio Hosomi, Tatsuru Okamura

**Affiliations:** 1 Department of Thoracic Oncology and Respiratory Medicine, Tokyo Metropolitan Cancer and Infectious Diseases Centre, Komagome Hospital, Honkomagome 3-18-22, Bunkyo, Tokyo, 113-0021 Japan; 2 Department of Pulmonary Medicine and Oncology, Graduate School of Medicine, Nippon Medical School, Tokyo, Japan

**Keywords:** Advanced non-small cell lung cancer, Chemotherapy, Nanoparticle albumin-bound paclitaxel, Later line setting

## Abstract

**Background:**

Few studies have investigated the clinical efficacy of third- and later-line of chemotherapy after standard chemotherapy for previously treated advanced non-small cell lung cancer (NSCLC). We prospectively evaluated the efficacy and safety of nanoparticle albumin-bound paclitaxel (nab-paclitaxel) following standard chemotherapies for previously treated advanced NSCLC.

**Methods:**

The eligible patients having adequate organ functions with performance status 0–2 were enrolled after completing standard chemotherapy. They received weekly nab-paclitaxel 100 mg/m^2^ intravenously on days 1, 8, and 15 every 3 weeks. The primary end point was objective response rate (ORR). Median progression-free survival (PFS), overall survival (OS), and adverse events (AEs) were evaluated as secondary end points.

**Results:**

This trial was discontinued because of late accrual. Twenty two patients were enrolled from April 2013 and February 2019. The total ORR was 22.7% [95% CI 7.8–45.4] and disease control rate (DCR) was 81.8% [95% CI 59.7–94.8]. Median PFS was 3.4 months [95% CI 2.3–4.1] and median OS was 7.4 months [95% CI 4.2–10.7]. Median follow-up interval was 6.7 months hematological AEs of Grade 3/4 included anemia (18%), leukopenia (18%), and neutropenia (32%), while the most frequent nonhematological AEs were fatigue (50%) and peripheral neuropathy (36.4%). Severe AEs related to treatment were observed in only one patient.

**Conclusion:**

Nab-paclitaxel may be a safe and effective later-line chemotherapeutic option for previously treated advanced NSCLC after standard of chemotherapies based on other trials.

## Introduction

Non-small cell lung cancer (NSCLC) is one of the most common cancers and prognosis remains poor [1]. Platinum doublet chemotherapy is currently an essential first-line therapy for NSCLC [2]. In addition, new molecular agents targeting epidermal growth factor receptor (EGFR), anaplastic lymphoma kinase (ALK), or ROS1 have dramatically improved NSCLC outcomes for patients with genetic alterations [3]. Immune checkpoint inhibitors (ICIs) are another new treatment option for NSCLC [3]. Recently, several phase III trials (Keynote 189, 407 and Impower 150) demonstrated that combination therapy with platinum doublet and ICI resulted in longer median overall survival (OS) for naive advanced NSCLC compared to platinum doublet therapy alone [4–6], and this combination therapy is now considered standard for NSCLC without targetable genetic alterations.

Anticancer agents like docetaxel, pemetrexed, erlotinib, and tegafur/gimeracil/oteracil (the S-1 regimen) are standard treatments for previously treated NSCLC [7–11]. Combination therapy with docetaxel and ramucirumab resulted in longer progression-free survival (PFS) in patients with platinum doublet-refractory NSCLC [12]. Recently, several phase III trials reported better efficacy of ICI treatment for platinum doublet-refractory advanced NSCLC compared to docetaxel [13–15]. Therefore, ICIs are now accepted as part of the standard regimen for advanced NSCLC.

Most advanced NSCLC patients receive several lines of treatment, but there are few prospective trials investigating the efficacy and safety of third- or later-line therapies [16]. Nanoparticle albumin-bound paclitaxel (nab-PTX) was developed as a novel cytotoxic agent with a targeted drug delivery system to improve the therapeutic index of paclitaxel [17]. As first-line treatment, carboplatin plus nab-PTX combination therapy demonstrated significantly higher objective response rate (ORR), a non-significant 1-month improvement in median OS, and lower toxicity than carboplatin plus paclitaxel [18]. In addition, previous phase II trials reported good efficacy and safety of weekly nab-PTX monotherapy as second line treatment for patients with platinum doublet-refractory NSCLC [19–21]. However, to our knowledge there has been no prospective trial assessing the efficacy and safety of nab-PTX as third- or later-line treatment.

Here, we present results of a single-arm phase II trial conducted at the Tokyo Metropolitan Cancer and Infectious Disease Center investigating the efficacy and safety of weekly nab-PTX monotherapy following standard chemotherapy for advanced NSCLC.

## Patients and methods

### Eligibility criteria

Patients consenting to later-line chemotherapy after standard therapy, with an Eastern Cooperative Oncology Group Performance Status (ECOG-PS) of 0–2, and with histologically or cytologically confirmed stage IIIB or IV NSCLC were eligible for enrollment. Patients who had received previous paclitaxel or nab-PTX treatment for NSCLC were excluded. At study onset in 2013, standard therapy was defined as chemotherapy including docetaxel and pemetrexed in patients with non-squamous cell lung cancer or docetaxel in patients with squamous cell lung cancer [8, 9]. The ICI nivolumab was approved by the Ministry of Health, Labor and Welfare of Japan in December 2015, so we revised the definition of standard therapy to include ICIs in February 2016. If the tumor exhibited a genetic alteration, such as EGFR mutation or ALK rearrangement, targeted therapy using a tyrosine kinase inhibitor (TKI) was also defined as standard therapy. Other eligibility criteria included adequate cardiac, hematologic, hepatic, renal, and respiratory function [oxygen saturation in room air ≥ 90%, hemoglobin content ≥ 9.0 g/dL, neutrophil count ≥ 1500/mm^3^, platelet count ≥ 100,000/mm^3^, aspartate aminotransferase (AST) and alanine aminotransferase (ALT) levels ≤ 2.5 times the upper limit of normal, total bilirubin concentration ≤ 1.5 mg/dL, and creatinine concentration ≤ 1.5 mg/dL] and life expectancy of more than 12 months. If patients had received prior radiotherapy or invasive therapy, such as chest drainage or pleurodesis, nab-PTX was not started for at least 2 weeks post-treatment. Other exclusion criteria included active concomitant malignancy without carcinoma in situ, uncontrollable central nervous system metastasis, uncontrollable pleural effusion, active infection, non-healing peptic wounds, severe complications of heart disease, interstitial pneumonia, uncontrolled hypertension, diabetes or other metabolic diseases, and drug sensitivity including to paclitaxel and albumin.

### Study design

This was a phase II, single-arm, single-center, open-label study of nab-PTX in patients with relapsed NSCLC after standard chemotherapy conducted at the Tokyo Metropolitan Cancer and Infectious Disease Center, Komagome Hospital (Tokyo, Japan). The primary endpoint was ORR. Secondary endpoints included PFS, OS, and adverse events (AEs) profile. The study protocol was approved by the Institutional Review Board of Tokyo Metropolitan Cancer and Infectious Diseases Centre, Komagome Hospital (#1212), and adhered to the tenets of the Declaration of Helsinki (World Medical Association). The study was registered with the UMIN Clinical Trials Registry (UMIN000010737). Informed consent was obtained from all participants.

### Treatment schedule

All patients received nab-PTX at 100 mg/m^2^ via intravenous infusion for 30 min on days 1, 8, and 15 of each 3-week cycle. Before the next treatment cycle, each patient was required to meet the following criteria: neutrophil count ≥ 1500/mm^3^, platelet count ≥ 75,000/mm^3^, hemoglobin content ≥ 9.0 g/dL, AST and ALT levels ≤ 2.5 times the upper limit of normal, total bilirubin concentration ≤ 2.0 mg/dL, creatinine concentration ≤ 2.0 mg/dL, and only tolerable nonhematologic AEs. On days 8 or 15 of each cycle, patients were also required to meet the following criteria: neutrophil count ≥ 1000/mm^3^, platelet count ≥ 50,000/mm^3^, peripheral neuropathy < grade 3, and ECOG PS ≤ 2. Dose reductions of 25 mg/m^2^ to a minimum dose of 50 mg/m^2^ were allowed for grade 4 neutropenia lasting longer than 7 days, thrombocytopenia grade 3 or 4, or any nonhematologic toxicity including peripheral neuropathy of grade 3 or 4. Any patient who required a third dose reduction was withdrawn from the study. In addition, a patient was withdrawn if the next cycle was not started 3 weeks from the end of the previous treatment cycle. Treatment cycles continued unless there was progressive disease or intolerable AEs, or if the patient refuses to continue treatment.

After protocol treatment completion, the next chemotherapy was not started until disease progression. However, any treatment (including ICIs) was allowed after disease progression.

### Treatment assessment

Baseline disease and response to therapy were assessed using the Response Evaluation Criteria in Solid Tumors version 1.1 (RECIST ver.1.1). Chest X-ray was conducted after every treatment cycle, and chest and abdominal computed tomographic scan (CT scan) was conducted routinely every 6 weeks. Whole-brain magnetic resonance imaging and isotope bone scan or positron emission tomography with 2-deoxy-2-[fluorine-18] fluoro-d-glucose integrated with computed tomography (^18^F-FDG PET/CT) were conducted for disease evaluation. Patients showing a complete response (CR) or a partial response (PR) received imaging examination as confirmatory evaluation after an interval of at least 4 weeks post-treatment. A patient was classified with stable disease (SD) if the response was confirmed and maintained for 6 weeks or longer after the start of the protocol treatment. Progress-free survival was defined as the time from study entry to disease progression or death by any cause, and OS as the time from study entry to death by any cause.

Evaluations of participants at baseline and during or following each cycle included physical examination, vital signs, ECOG-PS, and complete hematology and biochemistry profiles. After the first cycle, all factors were evaluated every week. AEs were categorized and graded during each cycle according to the National Cancer Institute Common Terminology Criteria for Adverse Events (CTCAE) version 4.0.

### Statistical analysis

Based on a previous report, we estimated the expected ORR as 15% and the lower limit of interest as 5%. As this research was designed to have a statistical power of 80% and type I error of 0.05, we calculated that 52 patients were required and planned enrolling 55 patients over 60 months with follow-up intervals of 12 months.

Median PFS and median OS were assessed using the Kaplan–Meier method and log-rank test. For median disease control rate (DCR) and ORR, 90% and 95% CIs were calculated using Fisher’s exact test. All statistical analyses were performed using EZR (Saitama Medical Centre, Jichi Medical University, Saitama, Japan), a graphical user interface for R (a modified R commander designed for biostatistics).

## Results

### Patient characteristics

The median follow-up interval was 7.1 months. Table 1 presents patient baseline characteristics. Although we expected 55 patients were enrolled in this trial, only 22/55 (40%) patients were treated that met enrollment criteria between April 2013 and March 2018 for late accrual. Median age of the patients was 65 years (range 30–79 years) and six patients were over 70 years old. Men accounted for the majority (16, 72.7%) and 11 patients (50.0%) had a performance status of 2 (poor). Thirteen patients (59.1%) had adenocarcinoma and nine (40.9%) had squamous cell carcinoma. Seventeen patients (77.3%) had received 3 or more prior treatments. Only two patients (9.1%) were never smokers and only one patient (4.5%) harbored an EGFR mutation or ALK rearrangement. All patients had received chemotherapy including docetaxel and six patients (27.3%) had received ICIs as previous therapy. Only one patient had not received prior chemotherapy using a platinum-containing regimen (due to advanced age).

**Table 1:** Patient’s characteristics

Total number	*n* = 22
Age	
Median	65
Range	(30–79)
≥ 70 years	6 (27.3%)
< 70 years	16 (72.7%)
Gender	
Male	16 (72.7%)
Female	6 (27.3%)
ECOG PS	
0	0 (0.0%)
1	11 (50.0%)
2	11 (50.0%)
Histology	
Adenocarcinoma	13 (59.1%)
Squamous cell carcinoma	9 (41.0%)
A number of prior therapies	
Median (range)	4 (3–7)
≥ 4th line	17 (77.3%)
< 4th line	5 (22.7%)
Smoking history	
Current or former smoker	20 (90.9%)
Never smoker	2 (9.1%)
EGFR mutation	
Wild type	13 (59.1%)
Mutant	1 (4.5%)
Unknown	8 (36.4%)
ALK rearrangement	
Wild type	13 (59.1%)
Positive	1 (4.5%)
Unknown	8 (36.4%)
Prior EGFR/ALK-TKIs	
Yes	5 (22.7%)
No	17 (77.3%)
Prior docetaxel	
Yes	22 (100.0%)
No	0 (0.0%)
Prior ICI	
Yes	6 (27.3%)
No	16 (72.7%)

*ECOG PS* Eastern Cooperative Oncology Group Performance Status, *EGFR *epidermal growth factor receptor, *ALK* anaplastic lymphoma kinase, *TKI* tyrosine kinase inhibitor, *ICI* immune checkpoint inhibitor

### Treatment delivery

The median number of chemotherapy cycles per patient was 4 (range 1–8 cycles) (Table 1). The median dose intensity of nab-PTX was 62.3 mg/m^2^/week. Eleven patients (50%) experienced dose delay, for which the most common reason was neutropenia (5/11, 45.5%). However, no patients required dose reduction.

### Treatment efficacy

**Fig. 1:**
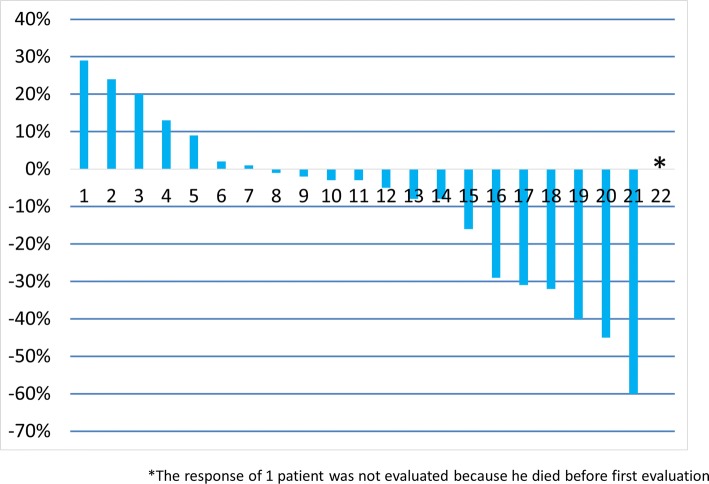
Response to nab-PTX presented as waterfall plot of greatest percentage change in target lesion size from baseline

**Fig. 2:**
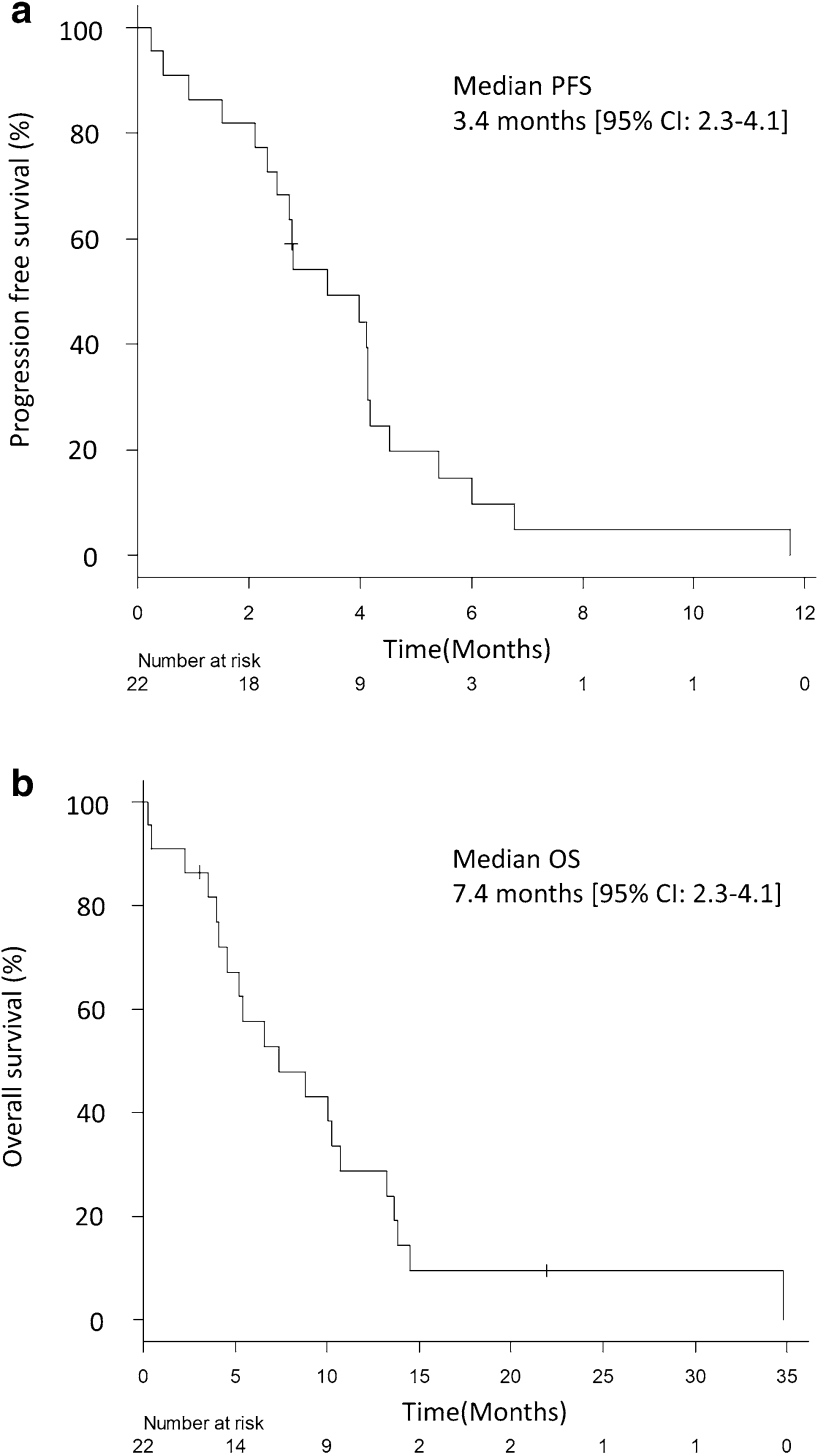
Kaplan–Meier curves for PFS (**a**) and OS (**b**) of all patients enrolled in this trial

Table 2 summarizes the treatment efficacy. Five patients achieved PR and 13 patients (59.1%) SD, while only three patients (18.2%) demonstrated progressive disease (PD). The total ORR was 22.7% [95% CI 7.8–45.4] and disease control rate (DCR) was 81.8% [95% CI 59.7–94.8]. Tumor response is presented in Fig. 1 as a waterfall plot. The response of one patient was not evaluated because he died before the first evaluation. Median PFS was 3.4 months [95% CI 2.3–4.1] (Fig. 2a) and median OS was 7.4 months [95% CI 4.2 − 10.7] (Fig. 2b).

**Table 2:** Response to weekly nab-PTX monotherapy after standard therapies

Response	Number of patients	%
CR	0	0.0
PR	5	22.7
SD	13	59.1
PD	3	13.6
NE	1	4.5
ORR	22.7 [95% CI 7.8–45.4]	
DCR	81.8 [95% CI 59.7–94.8]	

*CR* complete response, *PR* partial response, *SD* stable disease, *PD* progressive disease, *NE* not evaluable, *ORR* objective response rate, *DCR* disease control rate

### Safety

Profiles of major AEs are summarized in Table 3. Anemia was the most frequent AE (91%), including 18% with grade 3/4 anemia (18%). Other common hematological EAs were leukopenia (18%) and neutropenia (32%). Fatigue (50%) and peripheral neuropathy (36.4%) were the most frequent nonhematologic AEs. Although most AEs were generally mild and reversible, three patients (13.6%) experienced severe AEs (SAEs), including one case of severe thrombocytopenia requiring blood transfusion, one case requiring hospitalization for treatment of pneumonia unrelated to protocol treatment, and one death from cerebral stroke unrelated to protocol treatment. Febrile neutropenia and interstitial lung disease were not observed in this study.

**Table 3:** Toxicities of weekly nab-PTX monotherapy treatment (*N* = 22)

Toxicities	Any grade	%	Grade 3	%	Grade 4	%
Hematological						
Anemia	20	91	2	9	2	9
Thrombocytopenia	3	14	0	0	1	5
Leukopenia	16	73	4	18	0	0
Neutropenia	15	68	7	32	0	0
Febrile neutropenia					0	0
Non-hematological						
Nausea	2	9	0	0	0	0
Fatigue	11	50	5	23	0	0
Anorexia	6	27	0	0	0	0
Peripheral neuropathy	8	36	2	9	0	0
ALT/AST elevation	4	18	0	0	0	0
Rash	0	0	0	0	0	0
Interstitial lung disease	0	0	0	0	0	0

## Discussion

In this trial, we present the results of a phase II trial of weekly nab-PTX monotherapy after standard chemotherapy for advanced NSCLC. Previous prospective trials concluded that weekly nab-PTX monotherapy demonstrated a useful option for platinum-refractory NSCLC (as indicated by ORRs of 16.1–31.7%, DCRs of 65.9–71.9%, median PFS values of 3.9–4.9 months, and median OS values of 6.8–15.7 months) [19–21]. Tanaka et al. reported an ORR of 19.3%, DCR of 74.1%, median PFS of 4.5 months, and median OS of 15.7 months in a single-arm phase II study of weekly nab-PTX monotherapy for patients with chemorefractory NSCLC, including patients receiving third- or later-line treatment (32% of patients) [22]. Based on these trials, a phase III randomized clinical trial comparing nab-PTX to docetaxel in patients with platinum doublet-refractory advanced NSCLC is currently ongoing in Japan (UMIN00017487) [23]. However, there has been no prospective trial of weekly nab-PTX for advanced NSCLC exclusively as third- or later-line therapy (i.e., excluding weekly nab-PTX as first- or second-line treatment), although two retrospective studies have examined the efficacy of weekly nab-PTX monotherapy as later-line treatment for advanced NSCLC [24, 25]. Moreover, there is little data on the efficacy and safety of third- or later-line chemotherapy for advanced NSCLC. Harada et al. reported an ORR of 9.8%, median PFS of 3.0 months, and DCR of 61.0% for a single-arm phase II trial of amrubicin monotherapy as third- or fourth-line treatment [26]. On the other hand, a phase II trial of erlotinib vs. S-1 as a third- or fourth-line therapy in patients with advanced NSCLC reported an ORR of 16.7%, DCR of 66.7%, and PFS of 3.3 months for S-1 [27], while a retrospective study reported S-1 ORR and DCR values of 17.0% and 34.4%, respectively, as third-line treatment and 11.3% and 24.5%, respectively, as fourth-line treatment [16].

Weekly nab-PTX monotherapy demonstrated good safety and efficacy after standard therapy for advanced NSCLC despite use as later-line treatment and despite half of the patients having poor performance status (PS of 2), suggesting that nab-PTX may be effective at any time for advanced NSCLC. Moreover, ICI was one of the key drugs for first-line treatment of advanced NSCLC, so nab-PTX appears to be a useful treatment after ICI for advanced NSCLC patients. However, only six ICI-treated patients were included, so further studies assessing efficacy of chemotherapy after ICI treatment for advanced NSCLC are required.

The main AEs encountered in this trial were anemia, leucopenia, neutropenia, and peripheral neuropathy, while only three patients experienced severe AEs. Moreover, despite the inclusion of PS 2 patients, we found that efficacy and toxicity were equivalent to previous reports on weekly nab-PTX monotherapy. Thus, the AEs associated with weekly nab-PTX for advanced NSCLC are generally acceptable and manageable.

Weekly nab-PTX yielded a high responses rate despite inclusion of docetaxel as standard therapy for all patients. Cross-resistance to taxanes has been reported in preclinical studies and studies of breast cancer [28, 29]. However, analysis of TAX 317, TAX 320, and other trials revealed that prior paclitaxel treatment did not affect docetaxel efficacy [30]. On the contrary, few studies have examined the influence of prior docetaxel on nab-PTX treatment response. Our study suggests that such cross-resistance to taxanes is minimal.

This manuscript has several limitations. The key limitation is the smaller-than-expected number of patients meeting enrollment criteria within the study period. For this reason, the primary end point was obviously underpowered. Further, the long enrollment period may have affected the results given changes in early line regimens over time. Nonetheless, this is the first trial suggesting that nab-PTX is effective and safe as even third- or later-line treatment for advanced NSCLC following standard chemotherapies (including docetaxel). The clinical utility and safety of nab-PTX as third- or later-line treatment for advanced NSCLC may be shown in the current trial with the view of the possibility of cross-resistance for docetaxel, therefore, no more further larger-scale multicenter trials will be required to verify the effectiveness.

## Conclusion

Weekly nab-PTX monotherapy demonstrates good efficacy and only mild toxicity as later-line treatment after standard treatment for advanced NSCLC.
